# Refining filtering criteria of Kraken family of tools for accurate taxonomic profiling of ancient metagenomic data

**DOI:** 10.3389/fmicb.2026.1603339

**Published:** 2026-05-18

**Authors:** Nikolay Oskolkov

**Affiliations:** 1 Department of Biology, National Bioinformatics Infrastructure Sweden, Science for Life Laboratory, Lund University, Lund, Sweden; 2 Metabolic Research Group, Latvian Institute of Organic Synthesis, Riga, Latvia

**Keywords:** ancient DNA, ancient metagenomics, ancient pathogens, metagenomics, microbiome profiling

## Abstract

Taxonomic profiling is a key component of ancient metagenomic analysis, however it is also susceptible to false-positive identifications. In particular, taxonomic classification tools from the Kraken family, such as Kraken2 and KrakenUniq, are highly sensitive to the choice of filtering options. To address this issue, various filtering approaches have been proposed. In this study, I conduct a comprehensive benchmarking of different filtering strategies for Kraken family of tools using simulated microbial and environmental ancient metagenomic data. I evaluate these approaches based on the balance between sensitivity and specificity of ground truth reconstruction (F1-score), and propose an optimal thresholding strategy tailored to specific sequencing depths in ancient metagenomic datasets.

## Introduction

Metagenomic taxonomic profiling is a fundamental tool for studying both ancient and modern microbial communities, providing insights into past and present ecosystems. A variety of taxonomic classification tools, such as KrakenUniq (Breitwieser et al., 2018), Kraken2 (Wood et al., 2019), MetaPhlan (Blanco-Míguez et al., 2023), and Centrifuge (Kim et al., 2016), have been developed to analyze metagenomic datasets. However, selecting the most appropriate classifier remains a subject of debate, particularly in the field of ancient metagenomics, where challenges such as low coverage (limited amount of DNA), short read length, DNA degradation and modern contamination complicate taxonomic assignments.

Several benchmarking studies have systematically compared different metagenomic taxonomic classification approaches, assessing their accuracy, sensitivity, and specificity across diverse datasets (Velsko et al., 2018; Arizmendi Cárdenas et al., 2022; Pusadkar and Azad, 2023). These studies highlight the strengths and limitations of various tools, emphasizing factors such as database completeness, algorithmic differences, and computational efficiency. However, a critical question remains: whether the true performance of classification algorithms themselves, or simply the impact of post-classification filtering strategies has been compared? Since different tools employ distinct filtering thresholds and confidence scoring mechanisms, variations in performance may often be attributed to differences in downstream processing rather than fundamental differences in classification algorithms.

Thus, a more nuanced approach is required when interpreting benchmarking results, one that considers not only the taxonomic classifiers but also the filtering strategies applied to their outputs. Understanding these distinctions is essential for optimizing metagenomic taxonomic profiling, particularly in the context of ancient DNA studies, where accurate classification is crucial for distinguishing authentic ancient signals from modern contamination.

The Kraken family of tools (Breitwieser et al., 2018; Wood et al., 2019) generates highly detailed taxonomic profiling outputs, which can be overwhelming and difficult to interpret without appropriate filtering. Proper filtering significantly enhances the clarity of the results, yielding a more coherent and biologically meaningful list of identified organisms. In many cases, the filtered output aligns much better with expectations than the raw, unfiltered results. Kraken2 and KrakenUniq offer several key metrics that can be leveraged for filtering. One important metric is the number of reads assigned to each organism, which serves as a proxy for depth of sequencing coverage. Another crucial metric is the number of unique *k*-mers (number of unique/distinct minimizers in Kraken2) associated with an organism, which acts as a proxy for breadth of sequencing coverage. The latter is particularly important, as it helps eliminate false-positive read classifications caused by the miss-assignment of reads to highly conserved or convergently evolved genomic regions. This issue has been extensively discussed in previous studies (Breitwieser et al., 2018; Pochon et al., 2023), which highlight the risk of erroneous taxonomic identifications arising from sequence similarity in non-specific genomic regions. It was shown in the original KrakenUniq publication (Breitwieser et al., 2018) that “for the discovery of pathogens in human patients … a unique *k*-mer count threshold of 1000 eliminated many background identifications”. In addition, the thresholds of 1,000 unique *k*-mers (breadth of overage) and 200 assigned reads (depth of coverage) were elaborated in (Pochon et al., 2023) and used by default in the ancient metagenomic profiling software aMeta.

As an alternative filtering strategy, E-value, which represents the combination of KrakenUniq filters (specifically, E-value = (K/R) * C, where K is the number of unique *k*-mers, R is the number of reads assigned to a taxon and C is the *k*-mer coverage), was previously suggested for metagenomic taxonomic classification by Guellil et al. (2022) with the optimal threshold varying between 0.001 and 0.1 depending on the dataset. The E-value attempts to balance the number of unique *k*-mers (K) normalized by supporting them number of reads (R) and the *k*-mer coverage (C). If R is large but K is small, this indicates that many reads are hitting the same few *k*-mers repeatedly—a potential red flag for reads miss-classification. However, even if the absolute value for K is large (and R is small), low coverage (C) suggests those *k*-mers are clumped and not well-distributed, weakening the confidence in the detection. The equation for E-value was later modified by Borry (2022), by embracing the *k*-mer coverage into the double-exponential function, for a better distinction between true- and false-positive taxonomic assignments by KrakenUniq.

In this study, I perform a comprehensive benchmarking of different filtering approaches for Kraken family of tools using two simulated ancient microbial and one ancient environmental DNA datasets, the results of the benchmarking are also validated against three real metagenomic datasets. I compare the filtering thresholds suggested in (Breitwieser et al., 2018; Pochon et al., 2023) with the alternative filtering approaches (Guellil et al., 2022; Borry, 2022) and demonstrate that filtering with respect to the number of unique *k*-mers alone provides the optimal balance between sensitivity and specificity of organism detection in typical metagenomic samples, which is not outperformed by the alternative more complex filtering approaches (Guellil et al., 2022; Borry, 2022). I also suggest a simple scaling law for extrapolating the suggested filtering threshold (of 1,000 for the number of unique *k*-mers) to deeply sequenced metagenomic samples.

## Methods

To benchmark different filtering approaches of Kraken tools, I used three ancient metagenomic datasets, simulated using the gargammel tool (Renaud et al., 2017): (1) a regular microbial dataset, (2) a pathogen-enriched microbial dataset, and (3) an environmental (sedimentary) ancient DNA dataset. The first two datasets were adopted from (Pochon et al., 2023) and each consisted of 10 metagenomic samples (500,000 ancient and 500,000 modern DNA fragments simulated in each sample) with human as the host, mimicking the typical microbial composition observed in ancient Scandinavian human samples (Bergfeldt et al., 2024). The regular microbial dataset contained 35 microbial species (18 ancient and 17 modern) with microbial DNA percentage varied between 30 and 70%, while the pathogen-enriched dataset included 13 microbial species (9 ancient pathogens and 4 modern contaminants) with microbial reads occupying at most 30% of all reads. Full lists of included microbial species as well as the details of simulations are available in (Pochon et al., 2023). The heatmaps presenting the simulated ground truth for the the two microbial datasets are available as Supplementary Figure S5, S6, S18 in (Pochon et al., 2023).

Two ancient environmental (sedimentary) DNA (aeDNA) samples were simulated mimicking two opposite situations: (1) comparable DNA contribution from multiple organisms (vertebrates, invertebrates, plants and microbes), and (2) a single organism (presumed mammoth) dominated the DNA composition. In the first case, sixteen organisms across the tree of life (and across ecosystems, to avoid geographic biases in the data) were included: human (*Homo sapiens*), African elephant (*Loxodonta africana*), vesper bat (*Pipistrellus kuhlii*), wild boar (*Sus scrofa*), red junglefowl (*Gallus gallus*), saltwater crocodile (*Crocodylus porosus*), brown trout (*Salmo trutta*), thale cress (*Arabidopsis thaliana*), common sunflower (*Helianthus annuus*), black cottonwood (*Populus trichocarpa*), Australian freshwater crayfish (*Cherax quadricarinatus*), East Asian common octopus (*Octopus sinensis*), Old World swallowtail (*Papilio machaon*), and three microbial organisms: *Clostridium botulinum*, *Streptosporangium roseum* and *Yersinia pestis*. The primary motivation for selecting that diverse set of organisms, which would unlikely be present in a real sample, was to avoid any bias toward a particular group of organisms or ecosystem, and to ensure the inclusion of several negative controls for more robust analysis. In total, 252,498 ancient reads were simulated with 6.25% of reads originating from each of the sixteen species. The second simulated aeDNA sample contained 255,045 ancient reads with 30% being human (*Homo sapiens*), 7% microbial (*Clostridium botulinum*), and 63% of reads coming from African elephant (*Loxodonta africana*). This sample is assumed to mimic the situation of a very contaminated or degraded mammoth sample (van der Valk et al., 2021). The heatmaps presenting the simulated ground truth for the environmental/sedimentary ancient DNA samples are available in the Supplementary Figure S10.

Ancient reads for all samples were simulated with deamination following Briggs parameters (Jónsson et al., 2013; Briggs et al., 2007) in gargammel (Renaud et al., 2017): -damage 0.03, 0.4, 0.01, 0.3. The simulated ancient reads were fragmented and followed a log-normal distribution with the following parameters—loc 3.7424069808—scale 0.2795148843. These parameters were empirically determined from the *Y. pestis* reads in (Bergfeldt et al., 2024). Illumina sequencing errors were added with the ART module of gargammel (Renaud et al., 2017) to both modern and ancient reads. In addition, Illumina universal sequencing adapters were used, which resulted in 125 bp long paired-end reads.

Simulated ancient metagenomic reads were profiled by KrakenUniq and Kraken2 which employ a Lowest Common Ancestor (LCA) algorithm to handle DNA reads that map equally to multiple organisms. The databases for KrakenUniq and Kraken2 were built using the non-redundant NCBI NT database, which includes reference genomes from microbes, vertebrates, invertebrates, and plants. The default value *k* = 31 was used for the databases as it is known to provide sufficient specificity of organism detection in *k*-mer-based taxonomic classification (Arizmendi Cárdenas et al., 2022; Pochon et al., 2023). The database for both tools included identical reference genomes for consistency. The report outputs of KrakenUniq and Kraken2 were filtered using custom scripts, and an extensive benchmarking was conducted across a wide range of filtering thresholds.

In addition to benchmarking Kraken filters on three simulated datasets, six samples from three real datasets were used for validation. The organisms reported in the ans004 and stg001 samples (Bergfeldt et al., 2024), gok2c (Skoglund et al., 2014), and the adycha, krestovka, and blank samples (van der Valk et al., 2021) were treated as ground truth. To quantify the accuracy of ground truth reconstruction, the F1-score was calculated across different thresholds of unique *k*-mer counts.

**Figure 1:**
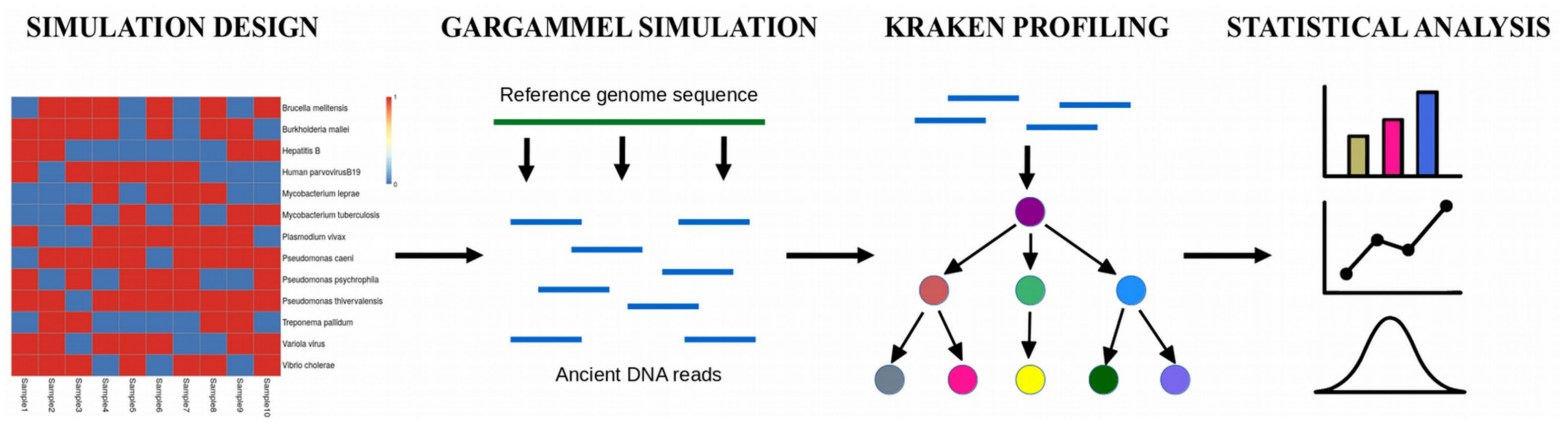
Overview of workflow used for benchmarking different filtering strategies of Kraken family of tools. Heatmaps of the simulated data: Red – organism present in the sample, blue – organism absent in the sample.

Figure 1 depicts a typical heatmap of simulated ground truth as well as the main steps of the benchmarking workflow used in this study.

## Results

**Figure 2:**
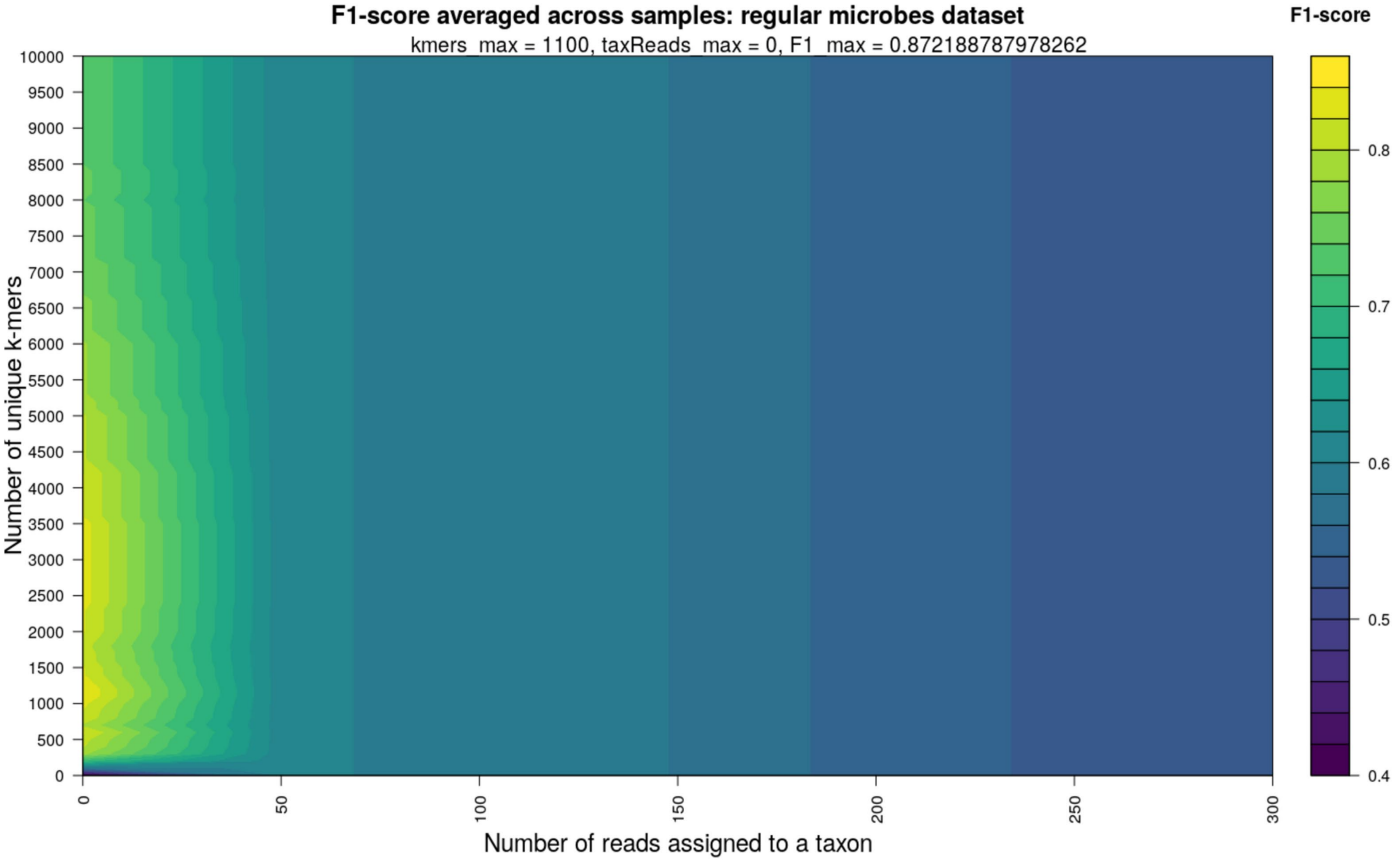
Heatmap of F1-score corresponding to ground truth reconstruction of regular microbial dataset for different values of KrakenUniq depth (number of reads assigned to a taxon) and breadth of coverage (number of unique *k*-mers) filters. The color gradient in the heatmap corresponds to F1-score values varying from 0 to 1. The highest value of F1 = 0.87 (brightest color) was achieved at 0 reads assigned to a taxon and ~1,000 unique *k*-mers.

To optimize the ground truth reconstruction by KrakenUniq, I explored a range of thresholds when simultaneously applying the depth (number of assigned reads) and breadth (number of unique *k*-mers) of coverage filters, and evaluating their impact on the F1-score—a metric in range from 0 to 1 balancing sensitivity and specificity of ground truth reconstruction. The 2D heatmap of F1-score (with the number of reads assigned to taxon as x- and the number of unique *k*-mers as y-coordinates) averaged across 10 simulated samples with regular microbial composition, presented in Figure 2, revealed that the number of unique *k*-mers alone was sufficient to achieve the highest F1-score, as no filtering with respect to the number of reads assigned to a taxon was needed. Specifically, a threshold of at least 1,000 unique *k*-mers and 0 reads assigned to a taxon provided optimal filtering, aligning with previously recommended values (Breitwieser et al., 2018; Pochon et al., 2023) for conservative microbiome profiling. The corresponding 2D heatmaps of F1-score computed for the microbial pathogen-enriched dataset and environmental/sedimentary aDNA dataset confirmed the thresholds of 1,000 and 1,500 unique *k*-mers, respectively, and 0 reads assigned to a taxon as nearly optimal for reconstructing the simulated ground truth, Supplementary Figure S1.

**Figure 3:**
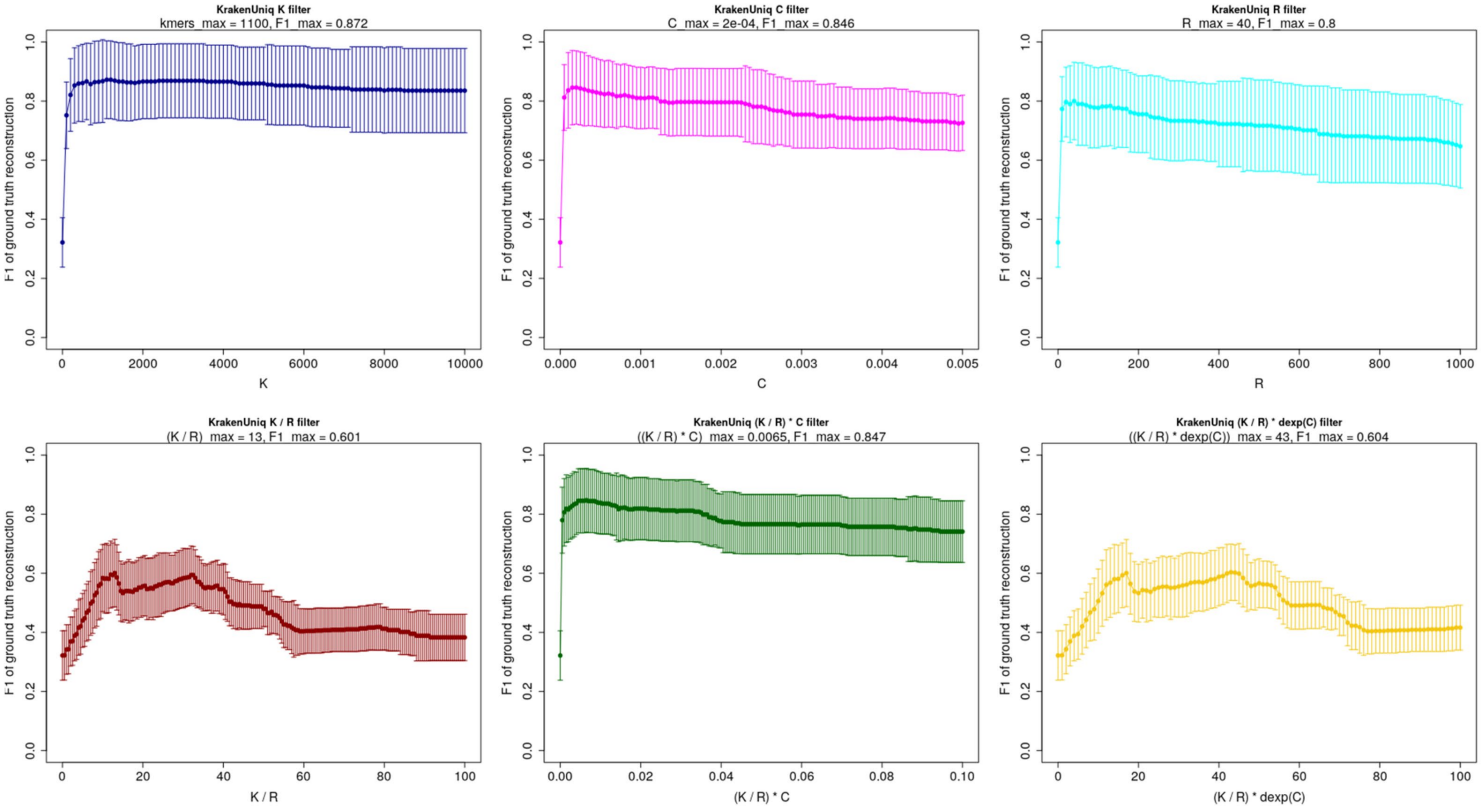
Comparison of different individual filtering approaches in terms of F1-score for simulated regular microbial dataset. The confidence intervals were computed by averaging across 10 samples.

I further examined how individual filtering metrics of Kraken family of tools affect the accuracy of taxonomic assignment. To achieve this, I explored a wide range of threshold values for six different Kraken filters: the number of unique *k*-mers (K filter), the number of assigned reads (R filter), the coverage of unique *k*-mers (C filter), the simple ratio of unique *k*-mers to assigned reads (K/R filter), the E-value-based filter (Guellil et al., 2022) (E-value = (K/R) * C filter), and a modified E-value filter according to (Borry, 2022) (E-value-modified = (K/R) * dexp(C) filter). Across all three simulated datasets and filtering strategies, the F1-score exhibited a sharp increase, reaching a peak before a gradual decline or almost a plateau (Figure 3, Supplementary Figures S2, S3). Despite the large error bars the overall profile suggests that a conservative approach with relatively stringent filtering thresholds is consistently more beneficial than a permissive approach, hence sharp growth of F1-score with the increase of filtering thresholds. This is explained by the fact that while less stringent filtering may improve sensitivity in detecting organisms within metagenomic samples, it also leads to a high number of false-positive identifications. On the other hand, a very conservative filtering does not seem to improve the ground truth reconstruction, and can even slightly worsen it, hence gradual decline or plateauing of F1-score at high filtering thresholds (Figure 3, Supplementary Figures S2, S3).

**Figure 4:**
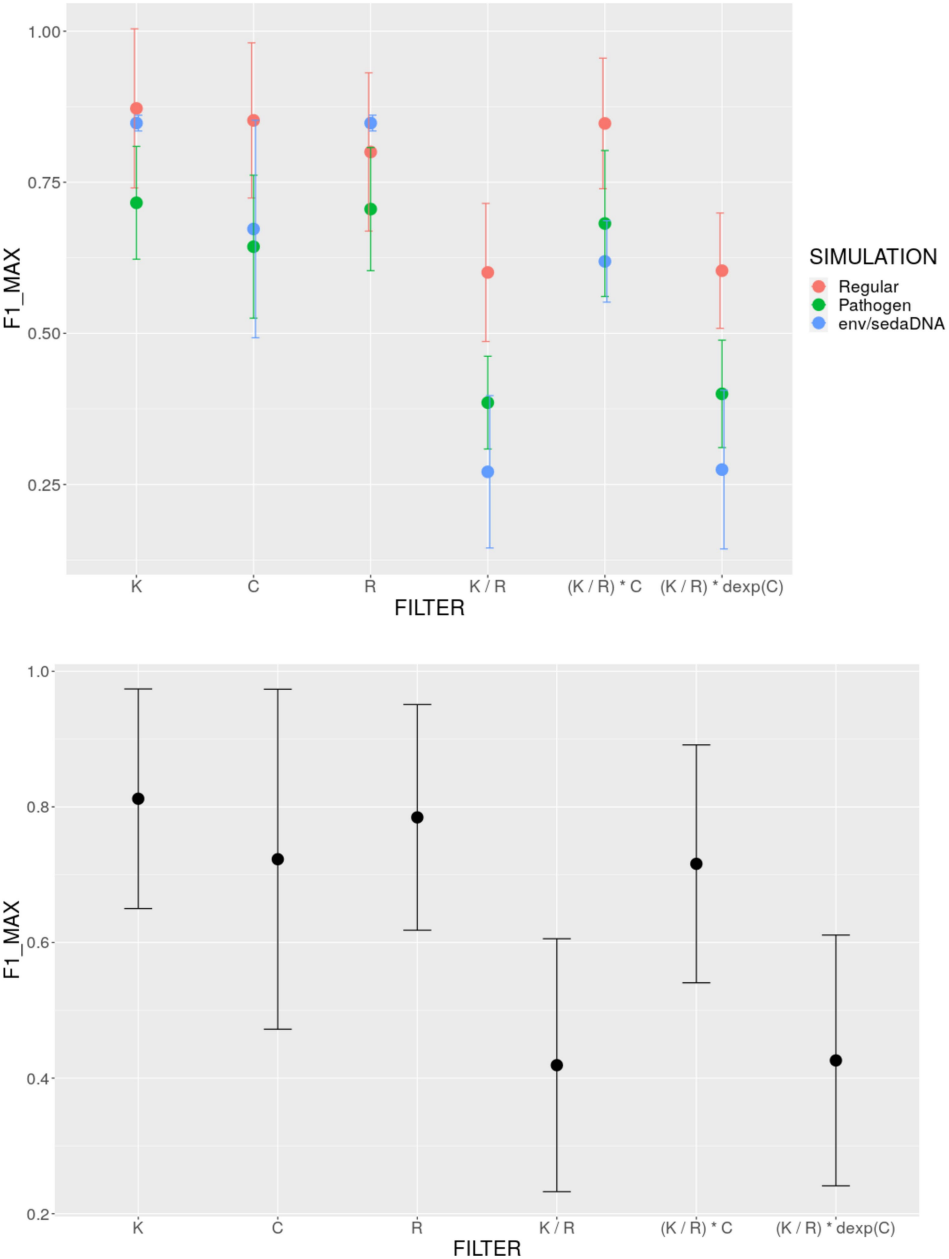
F1-score comparison of different filtering approaches of Kraken family of tools: (top) Three simulated datasets (regular microbial, pathogen-enriched microbial, and environmental/sedimentary ancient DNA) separately, (bottom) average across datasets.

When applying one Kraken filter at a time, the optimal number of unique *k*-mers was approximately 1,000 consistently across all three simulated datasets (Figure 3, Supplementary Figures S2, S3). Notably, a similar profile of F1-score peaking at 1,000 unique *k*-mers was also observed when validating on six metagenomic samples from three real datasets (Supplementary Figure S11), The optimal threshold for reads assigned to a taxon varied between 40 and 360, depending on the dataset (Figure 3, Supplementary Figures S2, S3). This aligns with previous findings (Pochon et al., 2023), which indicate that ~50 assigned reads serve as an absolute minimum for balancing sensitivity and specificity. However, for ancient DNA (aDNA) studies, slightly higher thresholds (~200 assigned reads) are generally recommended (Pochon et al., 2023). This is because obtaining a smooth deamination/damage profile with tools like mapDamage (Jónsson et al., 2013) becomes challenging when fewer than ~200 reads are available (Pochon et al., 2023). Among all the tested filters, the K-filter yielded the highest F1-score across all datasets: 0.87 for the regular microbiome dataset, 0.72 for the pathogen-enriched dataset, and 0.85 for the environmental DNA dataset. In contrast, the K/R filter and the modified *E*-value filter (Borry, 2022) consistently produced the lowest F1-scores across all three datasets. This trend is clearly illustrated in Figure 4, where I present the maximum F1-scores along with confidence intervals, averaged across all samples in each dataset. While the individual K, C, and R filters performed similarly in terms of F1-score (with the K-filter demonstrating a slight advantage), the combinations of filters, i.e., K/R and (K/R) * dexp(C) performed significantly worse (Kruskal-Wallis test *p* = 0.02, Dunn’s post-hoc test: *p* = 0.04 after Bonferroni correction), particularly for the environmental DNA dataset. However, the E-value filter (Guellil et al., 2022), defined as (K/R) * C, performed nearly as well as the individual K, R, and C filters for the regular and pathogen-enriched datasets. It is also important to note that while the K, R, and C filters yield comparable accuracy in reconstructing the ground truth, the E-value filter is much less interpretable and more difficult to grasp intuitively. In particular, it is unclear what level of genomic coverage the threshold values of 0.001–0.1 suggested in (Guellil et al., 2022), actually represent.

**Figure 5:**
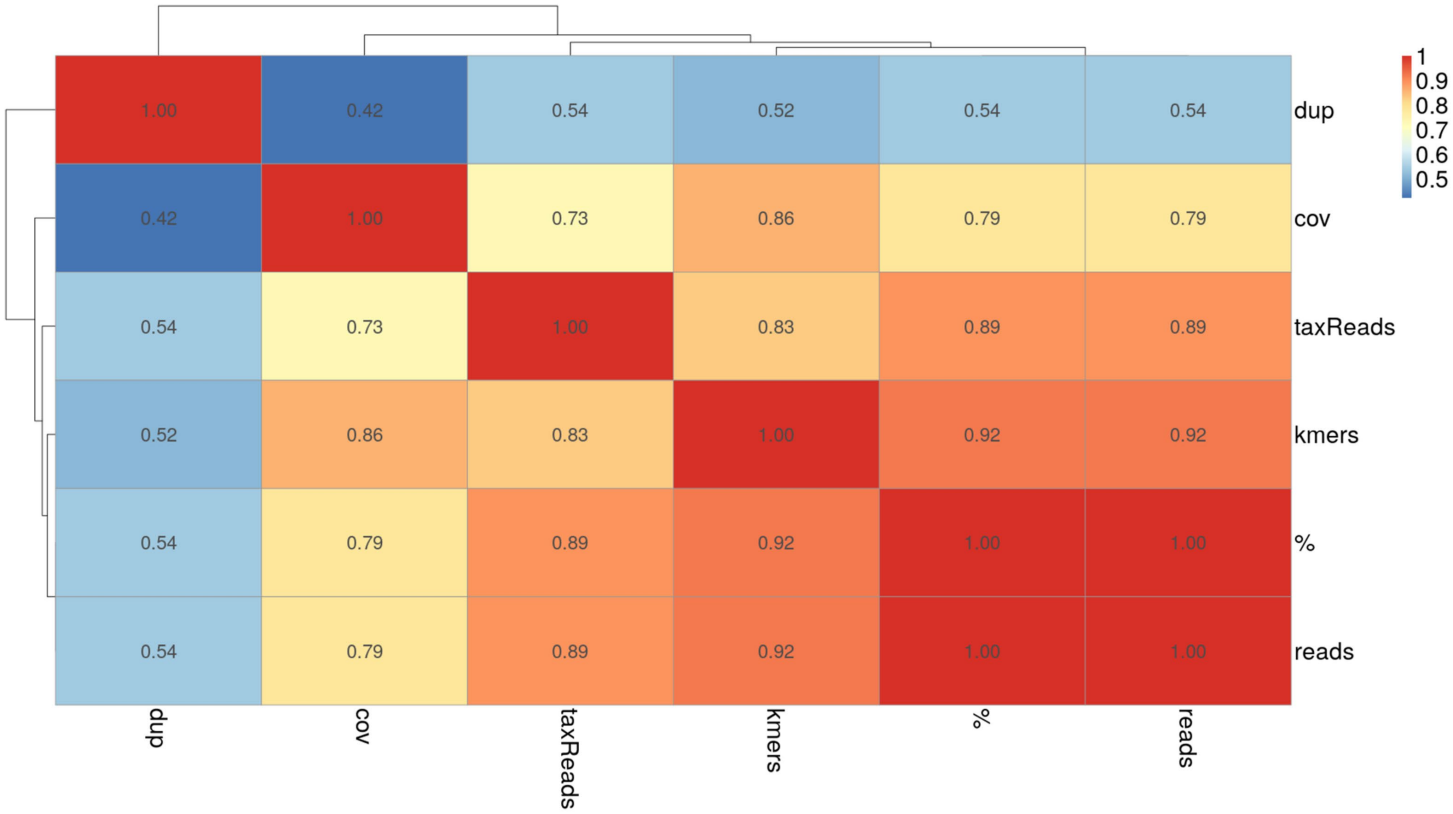
Pairwise Spearman correlation heatmap of KrakenUniq filters for regular microbial dataset averaged across all samples. Notations: % - percent of reads assigned to a clade, reads – number of reads assigned to a clade, kmers – number of unique *k*-mers, taxReads – number of reads assigned to a taxon, cov – coverage of the *k*-mers for a clade, dup – duplication level of *k*-mers.

To examine the relationships between different KrakenUniq filtering metrics, I generated a Spearman correlation heatmap for each dataset (Figure 5 and Supplementary Figures S4, S5). Overall, the heatmaps reveal a high degree of correlation among the metrics provided by KrakenUniq. In particular, the percentage of assigned reads, total number of reads assigned to a clade rooted in the taxon, number of reads assigned to a taxon, and the number of unique *k*-mers exhibit strong correlations, with Spearman’s rho values ranging from approximately 0.7 to 0.9. This finding helps explain why filtering based on either the number of unique *k*-mers or the number of assigned reads resulted in comparable F1-scores for reconstructing the ground truth (Figures 3, 4). In contrast, *k*-mer coverage and, more notably, the number of *k*-mer duplicates show weaker correlations with the other metrics, with Spearman’s rho values ranging from approximately 0.3 to 0.7.

Since Kraken2 and KrakenUniq employ different strategies for database construction and use slightly different breadth of coverage filters—KrakenUniq relies on the number of unique *k*-mers, while Kraken2 uses the number of unique/distinct minimizers—I aimed to compare their performances on regular and pathogen-enriched microbial datasets. To ensure a fair comparison, both tools were tested using databases containing identical reference genomes, and exhaustive filtering optimization was applied to their results. The key question was whether, given the most optimal filtering conditions for both KrakenUniq and Kraken2, their accuracy in reconstructing the ground truth would be comparable. Supplementary Figures S6, S7 show that, although numerically the optimal thresholds for unique *k*-mers are 2–3 times lower than the ones for unique/distinct minimizers, the resulting F1-scores are largely comparable, with KrakenUniq exhibiting slightly higher accuracy. Interestingly, Kraken2 appears to require an optimal number of unique/distinct minimizers that is at least 2–3 times higher than the optimal number of unique k-mers used by KrakenUniq.

Finally, I aimed at exploring whether deep sequencing of metagenomic samples can affect the number of unique *k*-mers threshold of 1,000 for KrakenUniq. I found that the optimal value of this threshold is significantly linearly associated (linear regression test *p* = 7*10^−7^, Pearson rho = 0.4, Spearman rho = 0.3) with sequencing depth and should be adjusted for deeply sequenced metagenomic samples. By determining the optimal number of unique *k*-mers that maximize the F1-score for ground truth reconstruction across 22 simulated samples, I observed that this optimal value increases approximately linearly with sequencing depth. Specifically, the relationship follows the approximate formula: optimal_n_unique_kmers ~ 0.002 * seq_depth. This translates to roughly 200 unique *k*-mers per 100,000 reads, as illustrated in Supplementary Figure S8. This simple scaling rule can be important to keep in mind when working with deeply sequenced metagenomic samples.

The relatively weak linear correlation between the optimal number of unique *k*-mers and sequencing depth, despite being statistically significant, is likely confounded by a few factors. First, it is important to note that Supplementary Figure S8 aggregates data from three datasets—regular microbial, pathogenic microbial, and environmental/sedimentary aDNA—each simulated for different purposes and under different assumptions. Specifically, there is a systematic difference in total sequencing depth between the regular microbial dataset (ranging from 300,000 to 700,000 reads) and pathogenic microbial dataset (ranging from 100,000 to 300,000 reads). Additionally, the simulated microbial communities differ in richness: 35 species were simulated in the regular dataset versus only 13 species in the pathogenic dataset. When analyzed separately, both datasets exhibit higher Spearman and Pearson correlation coefficients, ranging from 0.47 to 0.49. This confirms that the linear correlation between the optimal number of *k*-mers and sequencing depth is reproducible within each dataset. The lower correlation observed after aggregating the datasets is therefore likely attributable to batch effects or sub-optimal data harmonization.

Also, Supplementary Figure S8 shows signs of heteroscedasticity, which generally complicates statistical analysis. One possible explanation is the more pronounced plateauing of the F1 landscape in the regular microbial dataset (Figures 2, 3) compared to the pathogenic (Supplementary Figures S1, S2) and environmental/sedimentary aDNA (Supplementary Figures S1, S3) datasets. The wide plateau of the F1-score makes robust extremum detection challenging, resulting in greater uncertainty in identifying the position of the F1 maximum and, consequently, increased variation, i.e., higher heteroscedasticity.

The scaling law proposed in this study—“200 unique k-mers per 100,000 reads”—aligns closely with the analysis presented in the original KrakenUniq publication (Breitwieser et al., 2018), where the authors state in Figure 4: *“We found that the ideal thresholds increase by about 2000 unique k-mers per 1 million reads.”* While this suggests a similar scaling relationship, it is important to note that Breitwieser et al. based their conclusion solely on the Recall metric. In contrast, this study uses the F1-score, which balances both Precision and Recall, to determine the optimal scaling law, as shown in Supplementary Figure S8.

## Discussion

Taxonomic profiling of metagenomic data remains a cornerstone in profiling both past and modern prokaryotic and eukaryotic environments. The choice of an appropriate tool is a topic of ongoing debate in the field of ancient metagenomics, particularly as different methods employ distinct algorithms and heuristics. Several benchmarking studies have attempted to compare the performance of various taxonomic profilers, including Kraken2 and KrakenUniq, yet a consistent challenge lies in the fair assessment of these tools due to differences in how they filter their taxonomic profiling outputs (Velsko et al., 2018; Arizmendi Cárdenas et al., 2022; Pusadkar and Azad, 2023).

In this study, I systematically evaluated different filtering strategies of KrakenUniq and Kraken2 outputs using three simulated datasets: a regular microbial dataset, a pathogen-enriched microbial dataset, and an environmental ancient DNA (aDNA) dataset. My results suggest that filtering based on the number of unique *k*-mers is the most effective strategy for optimizing the accuracy of taxonomic assignment. This conclusion is evident from the Figure 2 which explores the effect of applying two filters simultaneously: the number of unique *k*-mers (K) and the number of reads assigned to a taxon (R). However, as consistently shown across the three simulated datasets (Supplementary Figure S1), the optimal filtering thresholds for maximizing the F1-score correspond to K > 1,000 and R ≈ 0. This indicates that filtering based on the number of assigned reads is largely unnecessary—very few (even close to zero) reads are sufficient, provided the number of unique *k*-mers is high. In other words, the two-dimensional filtering problem effectively reduces to a one-dimensional problem, as the contribution of R is negligible and, when enforced, may even reduce the accuracy of reconstructing the ground truth. Therefore, the filtering strategy advocated in this study is to rely solely on the number of unique *k*-mers, while keeping the read count filter very permissive (e.g., allowing as few as 1–5 reads). One still may want to increase the latter for authentication purposes, e. g. ancient DNA damage pattern computation with mapDamage (Pochon et al., 2023).

Note that, in contrast to the two-dimensional filtering shown in Figure 2—where two filters are applied simultaneously—Figure 3 examines one-dimensional filtering, where only a single filter is applied at a time. If filtering is based solely on the number of assigned reads (R-filter), an optimal threshold falls between approximately 40 and 360 reads (Figure 3). However, when both R and K (number of unique *k*-mers) filters are combined, the K filter clearly dominates in terms of its impact on the F1-score, and the R filter adds little to no benefit (Figure 2). In fact, including an R filter alongside K may reduce the accuracy of ground truth reconstruction. This suggests that multi-dimensional filtering using all KrakenUniq metrics is likely not advantageous. Instead, the K filter alone is sufficient and more effective for achieving optimal classification performance. Nonetheless, if only a single metric other than the K-filter is used for filtering (e.g., R or C), as in the one-dimensional analysis shown in Figure 3, then a moderate threshold should still be applied to maximize reconstruction accuracy.

It is important to emphasize that unlike other filtering approaches, such as those based on the number of assigned reads (R) or combined filtering metrics (e.g., E-value), the unique *k*-mer filter provided the highest F1-scores for ground truth reconstruction consistently across all three simulated datasets (Figure 4). This finding simplifies the filtering process and supports the use of unique *k*-mers as a reliable metric for reducing false-positive assignments in metagenomic studies.

Furthermore, my comparison of Kraken2 and KrakenUniq demonstrates that, although both tools apply different database construction strategy (on identical reference genomes), they yield comparable accuracy when optimized filtering parameters are applied. Although KrakenUniq relies on the number of unique *k*-mers, while Kraken2 utilizes the number of unique/distinct minimizers, my results indicate that, after exhaustive optimization, their performance differences are minimal. This highlights the importance of proper filtering rather than the choice of the taxonomic profiler itself. Interestingly, Kraken2 required 2–3 times more unique/distinct minimizers to achieve a comparable level of accuracy to KrakenUniq, suggesting that the two taxonomic classifiers differ in their sensitivity to sequence complexity and taxonomic resolution. The higher number of unique/distinct minimizers required to maintain the same level of accuracy as unique *k*-mers is likely due to the fact that a minimizer represents a substring of a *k*-mer, therefore multiple different *k*-mers can share the same minimizer, implying more minimizers are needed to achieve the same resolution as when using *k*-mers.

Another key finding of this study is that the optimal number of unique *k*-mers increases approximately linearly with the sequencing depth of metagenomic samples, Supplementary Figure S8. Specifically, in this study I derive a simple scaling law indicating that the optimal threshold for unique *k*-mers is approximately 200 per 100,000 reads. This relationship provides a practical guideline for researchers working with metagenomic datasets of varying sequencing depths, ensuring that filtering thresholds are appropriately adjusted to maintain accuracy without overly conservative exclusion of true positives.

It is also important to discuss that when two taxa, A and B, differ significantly in abundance, the ability of Kraken-tools to detect both is constrained by the total sequencing depth (in this study: the total number of simulated reads). For example, if both taxa are present but one is much more abundant than the other, the more abundant taxon is likely to be detected (using the recommended filtering thresholds from this study), while the less abundant one might be missed at low sequencing depth but successfully detected at higher depth. In this article I suggest that improving detection sensitivity should be achieved by increasing sequencing depth rather than loosening filtering thresholds, as more permissive filtering may compromise specificity and lead to false positives. In other words, reducing Kraken’s filtering thresholds with the aim of capturing both A and B can increase the risk of falsely detecting taxa that are not actually present. This is precisely the point illustrated in Supplementary Figure S8, where the “optimal number of unique *k*-mers” is defined as the threshold that maximizes the F1-score, balancing sensitivity and specificity of organism detection.

This study does not aim to provide a comprehensive benchmarking of all available taxonomic profilers in the field of ancient metagenomics. Conducting such a benchmarking would be highly challenging, as the tools are based on fundamentally different principles, filters, and profiling strategies. For example, alignment-based methods [e.g., BWA (Li and Durbin, 2009), Bowtie2 (Langmead and Salzberg, 2012) or MALT (Pochon et al., 2023)] differ significantly from the *k*-mer-based approaches and are not easily comparable to Kraken in terms of how they assign reads to references. Next, MetaPhlAn (Blanco-Míguez et al., 2023) represents a class of marker gene-based tools that rely on a fixed database heavily enriched for species associated with the human (e.g., gut) microbiome. This is fundamentally different from the genome-wide tools like Kraken and limits MetaPhlAn’s utility in ancient metagenomics, where DNA amounts are typically low and marker gene-based approaches lack the sensitivity needed to detect all the diversity of organisms, particularly those from less-studied environments such as soil or marine ecosystems. CLARK (Ounit et al., 2015) is perhaps the only *k*-mer-based tool somewhat comparable to Kraken. However, CLARK is not widely adopted within the ancient metagenomic community because its database construction excludes non-unique *k*-mers, leading to reduced sensitivity in detecting organisms with high genomic similarity. Also, CLARK is missing the unique *k*-mers metric which is absolutely critical for benchmarking against the Kraken family of tools. An additional reason why Kraken-based tools dominate current ancient metagenomic research is their computational efficiency and flexibility in building custom databases, which makes them highly adaptable to the increasing resource and diversity demands of the field.

Finally, it is important to mention that post-mortem DNA damage is an important authentication criterion for identifying and excluding modern contaminants from ancient metagenomic samples. However, the focus of this study is on detection rather than authentication filtering criteria. For a comprehensive comparison of detection versus authentication error in ancient metagenomic analyses, please refer to our previous publication of the aMeta workflow (Pochon et al., 2023). Technically, post-mortem DNA damage can confirm the ancient origin of sequenced DNA reads but cannot verify the accuracy of their taxonomic assignment. In other words, reads can indeed be ancient but still incorrectly assigned to an organism or mapped to the wrong reference genome, potentially leading to incorrect conclusions. The overarching aim of this study is to ensure accurate detection of organisms, regardless of whether the DNA is ancient or modern.

Despite these insights, several limitations should be considered. First, my benchmarking was conducted on simulated datasets, which, while valuable for controlled comparisons, may not capture the full complexity of real-world metagenomic samples, particularly in the presence of sequencing artifacts or uncharacterized microbial diversity. However, an accurate validation of the filtering capabilities of Kraken-tools on real datasets is problematic because their true composition is unknown. Nevertheless, one can assume that the organisms published in other studies represent the ground truth, this is attempted in the Supplementary Figure S11 which presents the binary heatmap (present vs. absent) for a few organisms (human, mammoth, microbes) for six samples published in three studies (Bergfeldt et al., 2024; Skoglund et al., 2014; van der Valk et al., 2021) together with the F1-score of their reconstruction at different filtering thresholds with respect to the number of unique *k*-mers. Although the large error bars highlight the high uncertainty in the composition of the real samples, the overall trend—a gradual increase in F1-score with mild filtering, peaking, and then declining/plateauing at higher unique *k*-mer thresholds—mirrors the pattern observed in Figure 3 and Supplementary Figures S2, S3 for simulated microbial and environmental/sedimentary aDNA samples. Notably, the highest F1-score is again achieved at the threshold of 1,000 unique *k*-mers, further supporting the robustness of this study’s conclusions and their potential applicability to real-world datasets.

Second limitation is that this study focuses exclusively on the Kraken family of tools for exploring different filtering approaches. The objective of this work was not to benchmark KrakenUniq and Kraken2 against other taxonomic profilers for two main reasons: (1) accurate benchmarking is non-trivial, as different tools apply filters in ways that are not directly comparable, making this task beyond the scope of the present study; and (2) the Kraken family remains one of the most flexible (e.g., allowing custom databases), powerful, and widely used tools in ancient metagenomic analysis, yet it still lacks systematic evaluation of possible filtering strategies. Therefore, the aim of this study was to provide clear and accurate best-practices recommendations for using Kraken-based tools within the ancient metagenomics community.

Third, another potential limitation of this study is the database choice. Indeed, since the NCBI NT database is an uncurated collection of references with varying quality, it can introduce biases due to unequal detection power for organisms with high-quality versus poor-quality reference genomes. However, the microbial component of NCBI NT, which is the primary focus of this study, largely mirrors the well-curated NCBI RefSeq references and, in this respect, should not introduce major biases. Furthermore, in contrast to other tested databases, NCBI NT demonstrated a strong ability to detect nearly all simulated organisms when reconstructing the ground truth in simulated environmental and sedimentary DNA metagenomic samples, as shown in the Supplementary Figure S9. Therefore, the main strength of NCBI NT and motivation to use in this study is its superior diversity (compared to other available databases) and ability to mitigate discovery biases of organisms from very different groups across the tree of life.

In conclusion, this study examines in details different filtering strategies of Kraken family of taxonomic profilers and provides strong evidence that filtering by the number of unique *k*-mers is the optimal strategy for taxonomic classification in ancient metagenomics. By applying this approach, researchers can enhance the accuracy of microbial and environmental profiling while minimizing false-positive taxonomic assignments. Additionally, the comparable performance of KrakenUniq and Kraken2 underscores the importance of filtering optimization over taxonomic classifier selection. These findings contribute to the ongoing refinement of best practices in metagenomic data analysis and offer practical guidelines for filtering strategies that can be adapted to diverse sequencing depths and study designs.

## Data Availability

The datasets presented in this study can be found in online repositories. The names of the repository/repositories and accession number(s) can be found in the article/Supplementary material.
