# Supplementary material for: Refining filtering criteria of Kraken family of tools for accurate taxonomic profiling of ancient metagenomic data
Source: Front Microbiol. 2026 May 18;17:1603339. doi: 10.3389/fmicb.2026.1603339 (PMC13223122; doi:10.3389/fmicb.2026.1603339)
Supplement: Supplementary file 1 [file Supplementary_file_1.pdf]

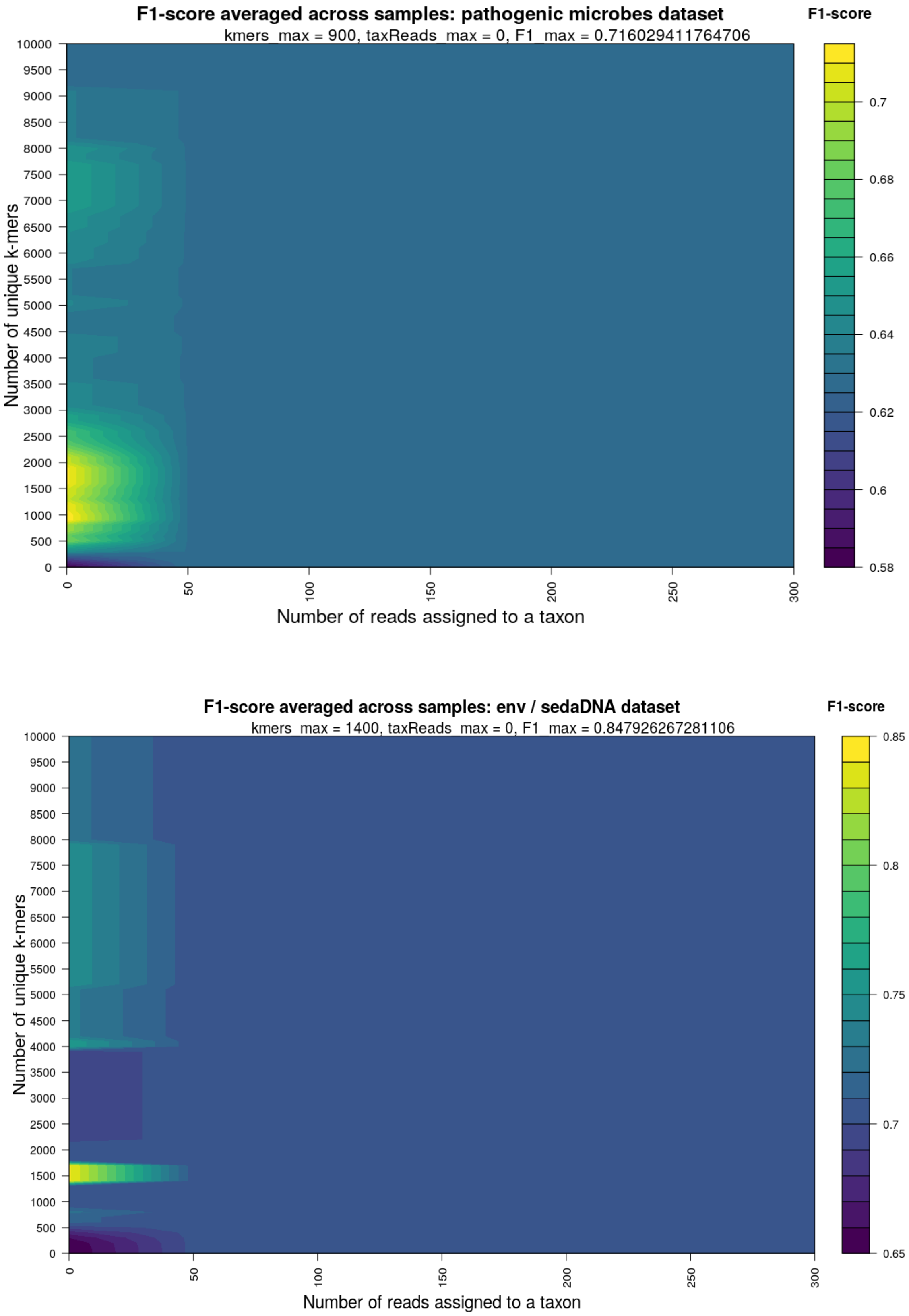

Supplementary Figure 1. Heatmap of F1-score of ground truth reconstruction for a range of KrakenUniq filters: microbial pathogen-enriched (top), and environmental / sedimentary aDNA (bottom) datasets. The color gradient corresponds to F1-score values varying from 0 to 1.

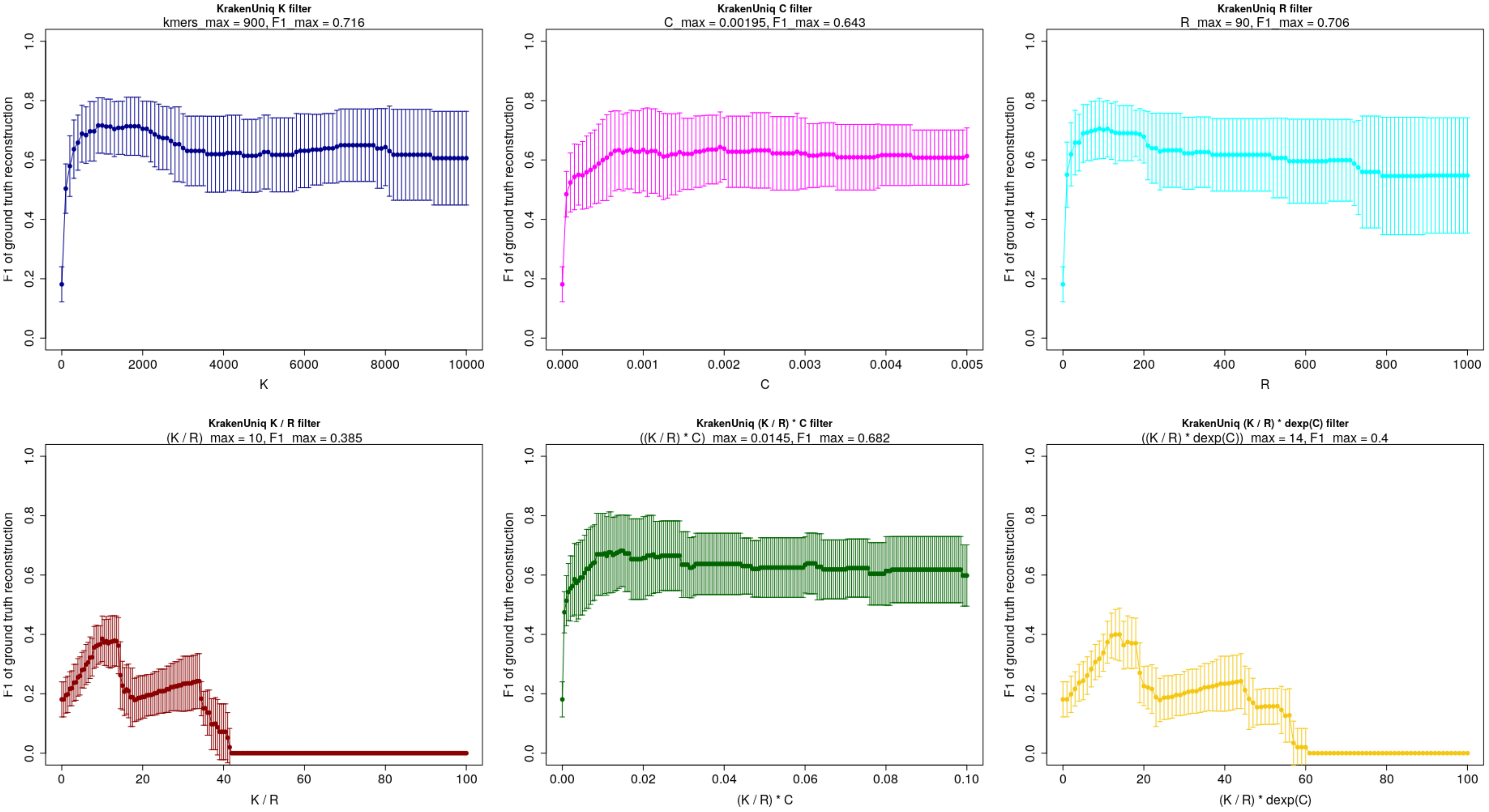

Supplementary Figure 2. Comparison of different filtering approaches in terms of F1 score for simulated microbial pathogen-enriched dataset.

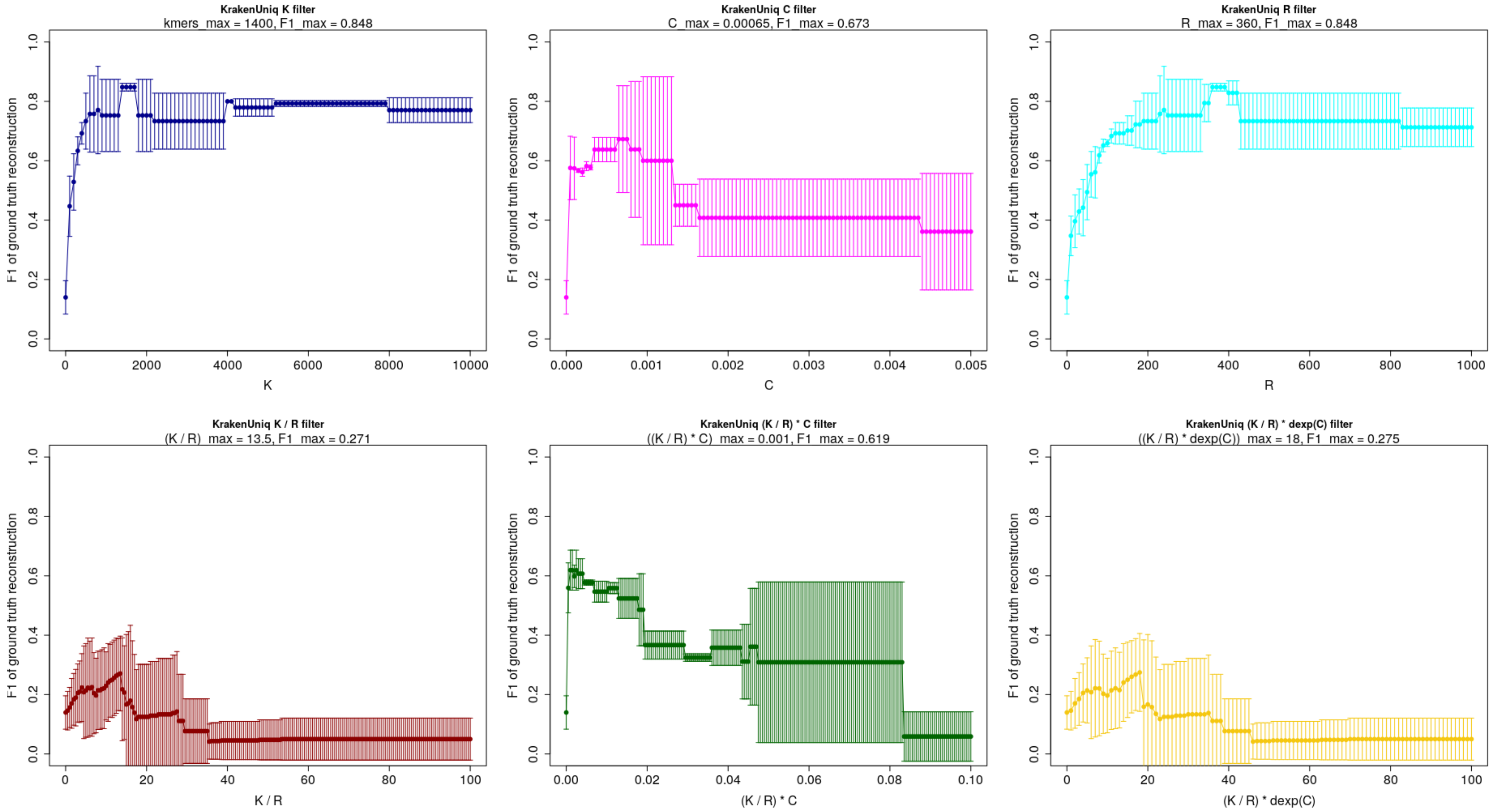

Supplementary Figure 3. Comparison of different filtering approaches in terms of F1 score for simulated environmental / sedimentary DNA dataset.

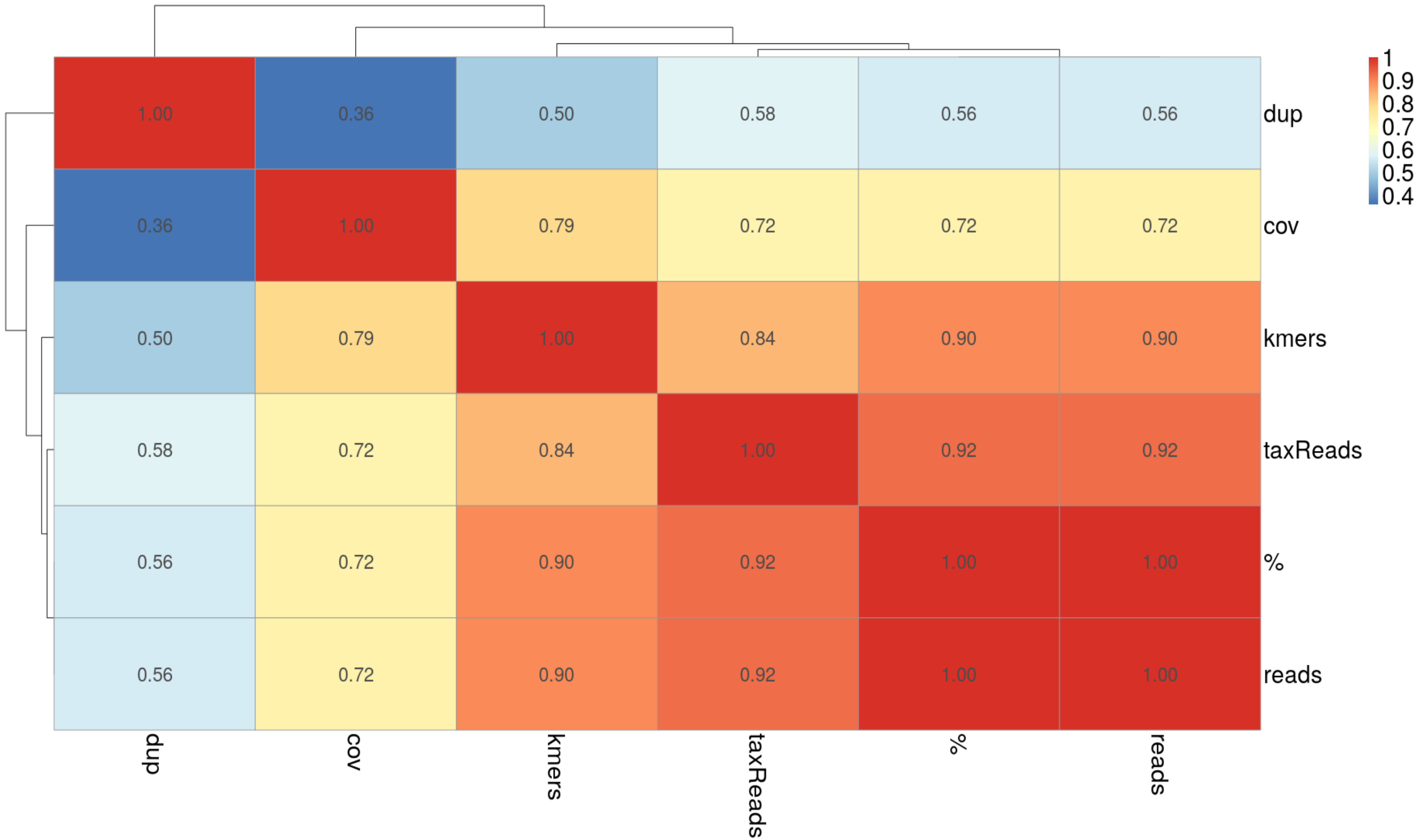

Supplementary Figure 4. Pairwise Spearman correlation heatmap of KrakenUniq filters for pathogen-enriched microbial dataset. Notations: % - percent of reads assigned to a clade rooted in the taxon, reads – number of reads assigned to a clade rooted in the taxon, kmers – number of unique *k*-mers, taxReads – number of reads assigned to a taxon, cov – coverage of the *k*-mers for a clade rooted in the taxon, dup – duplication level of *k*-mers.

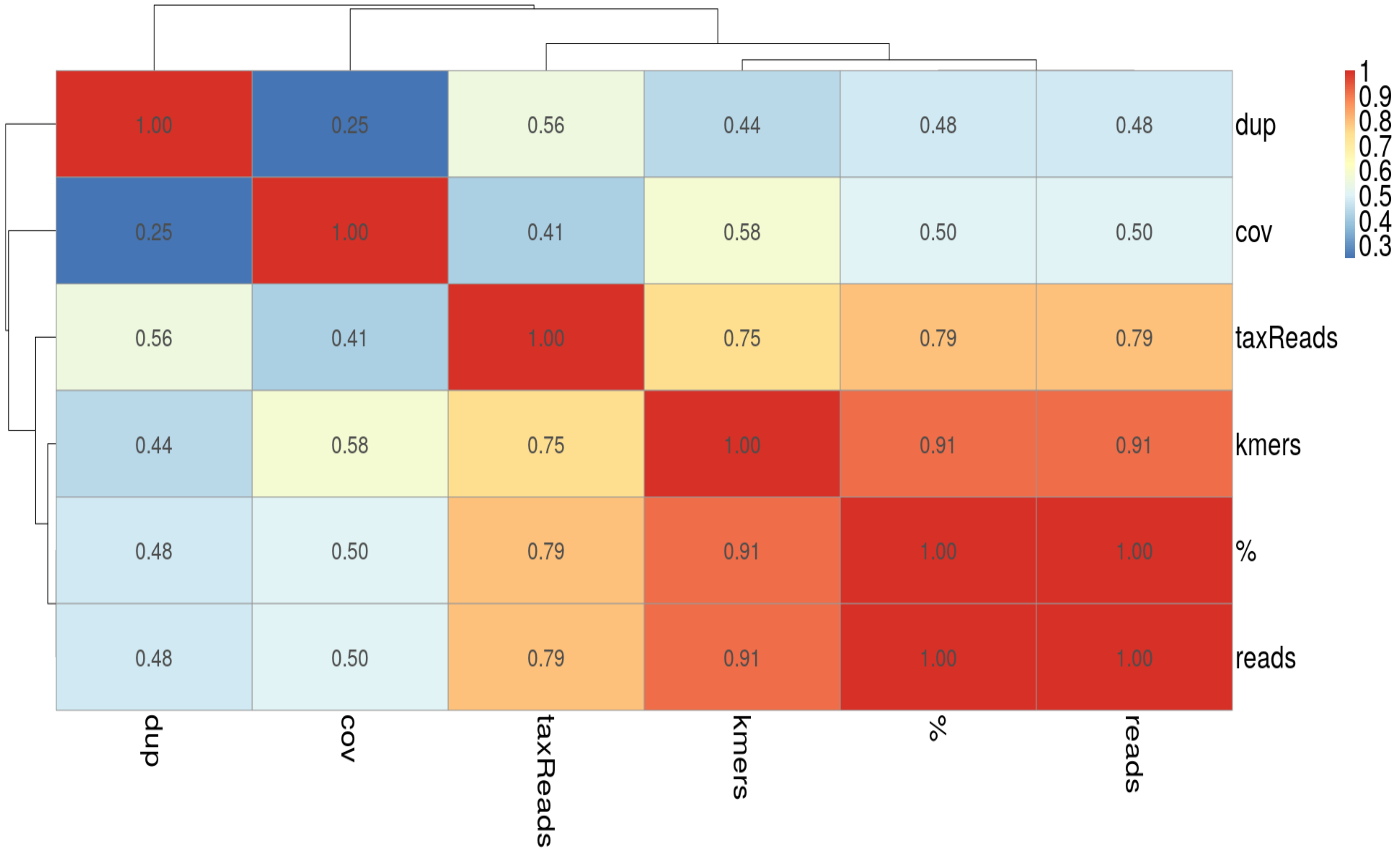

Supplementary Figure 5. Pairwise Spearman correlation heatmap of KrakenUniq filters for env / sedaDNA dataset. Notations: % - percent of reads assigned to a clade rooted in the taxon, reads – number of reads assigned to a clade rooted in the taxon, kmers – number of unique *k*-mers, taxReads – number of reads assigned to a taxon, cov – coverage of the *k*-mers for a clade rooted in the taxon, dup – duplication level of *k*-mers.

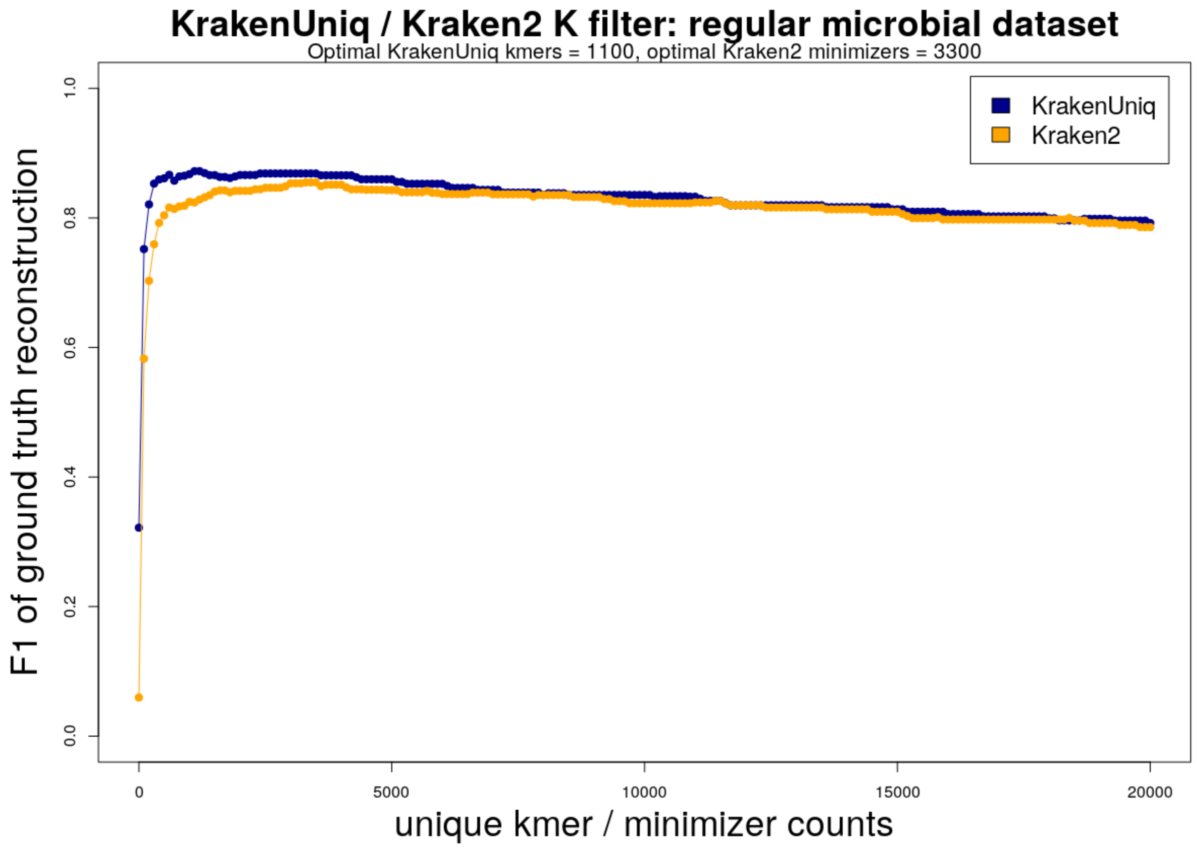

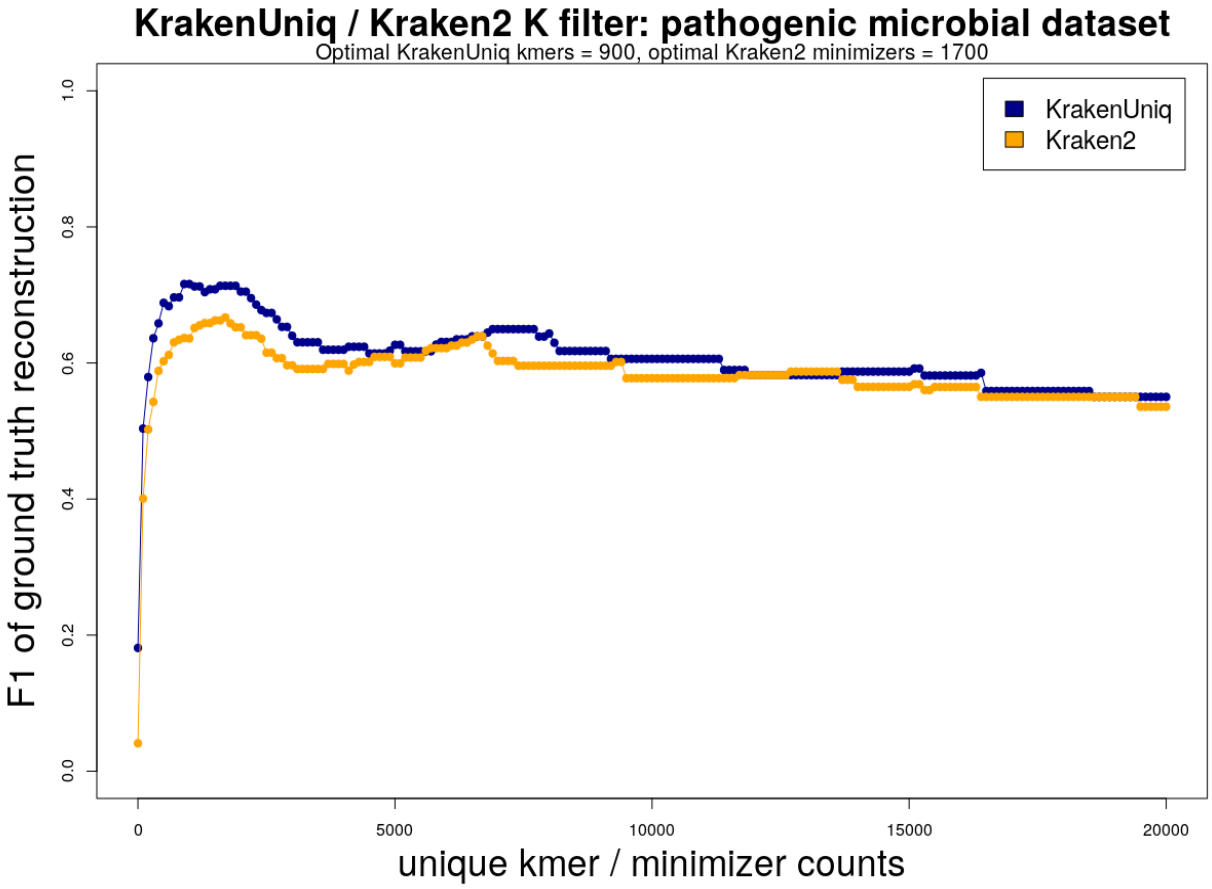

Supplementary Figure 6. Comparison of KrakenUniq vs. Kraken2 F1-score of ground truth reconstruction: regular (top), and pathogen-enriched (bottom) microbial datasets. In both case, the plots correspond to optimal filtering strategies applied to KrakenUniq and Kraken2 outputs.

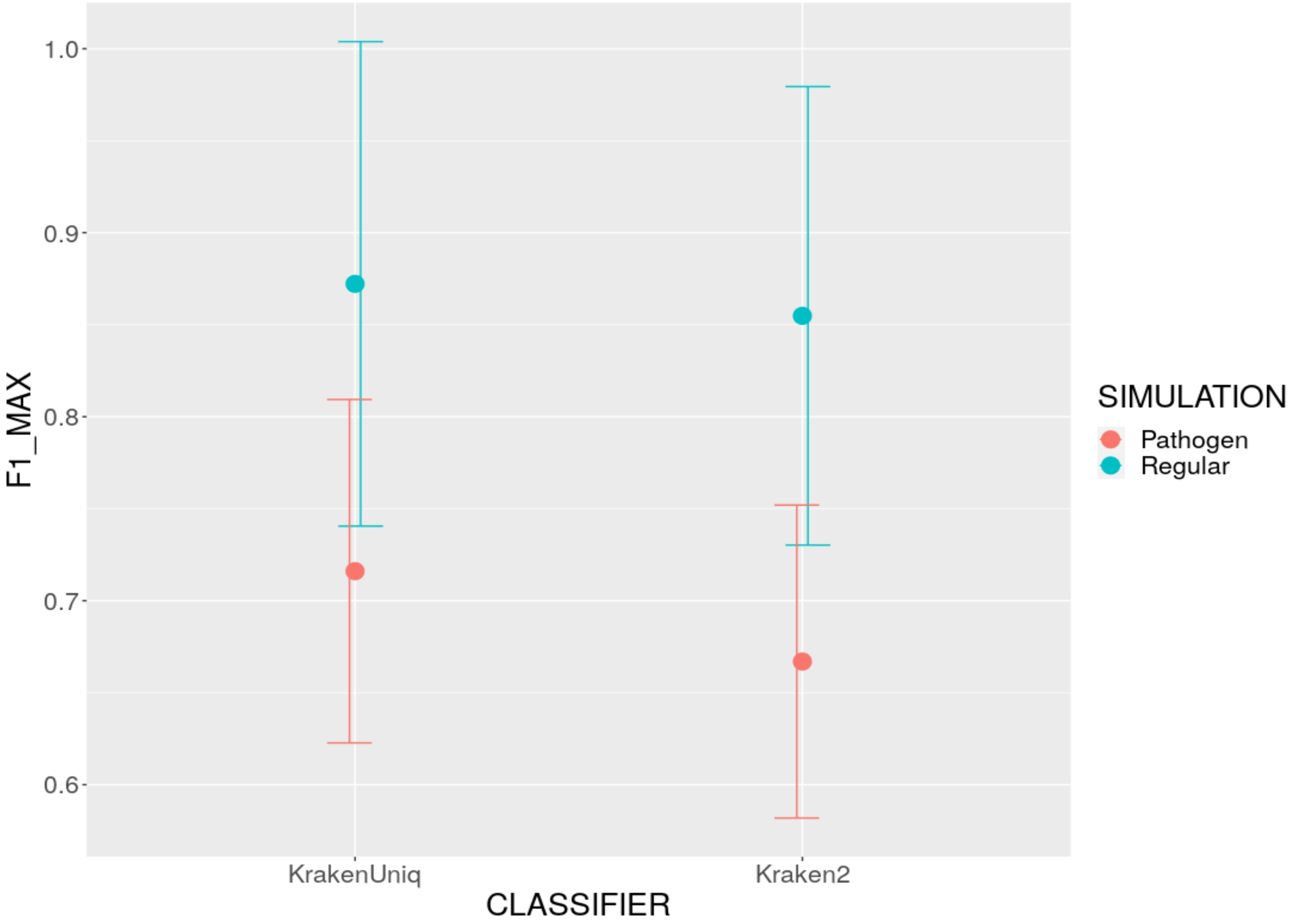

Supplementary Figure 7. Comparison of optimal F1-scores for KrakenUniq vs. Kraken2 performance on a) regular microbial, and b) pathogen-enriched microbial datasets. The difference in F1-score is not statistically significant.

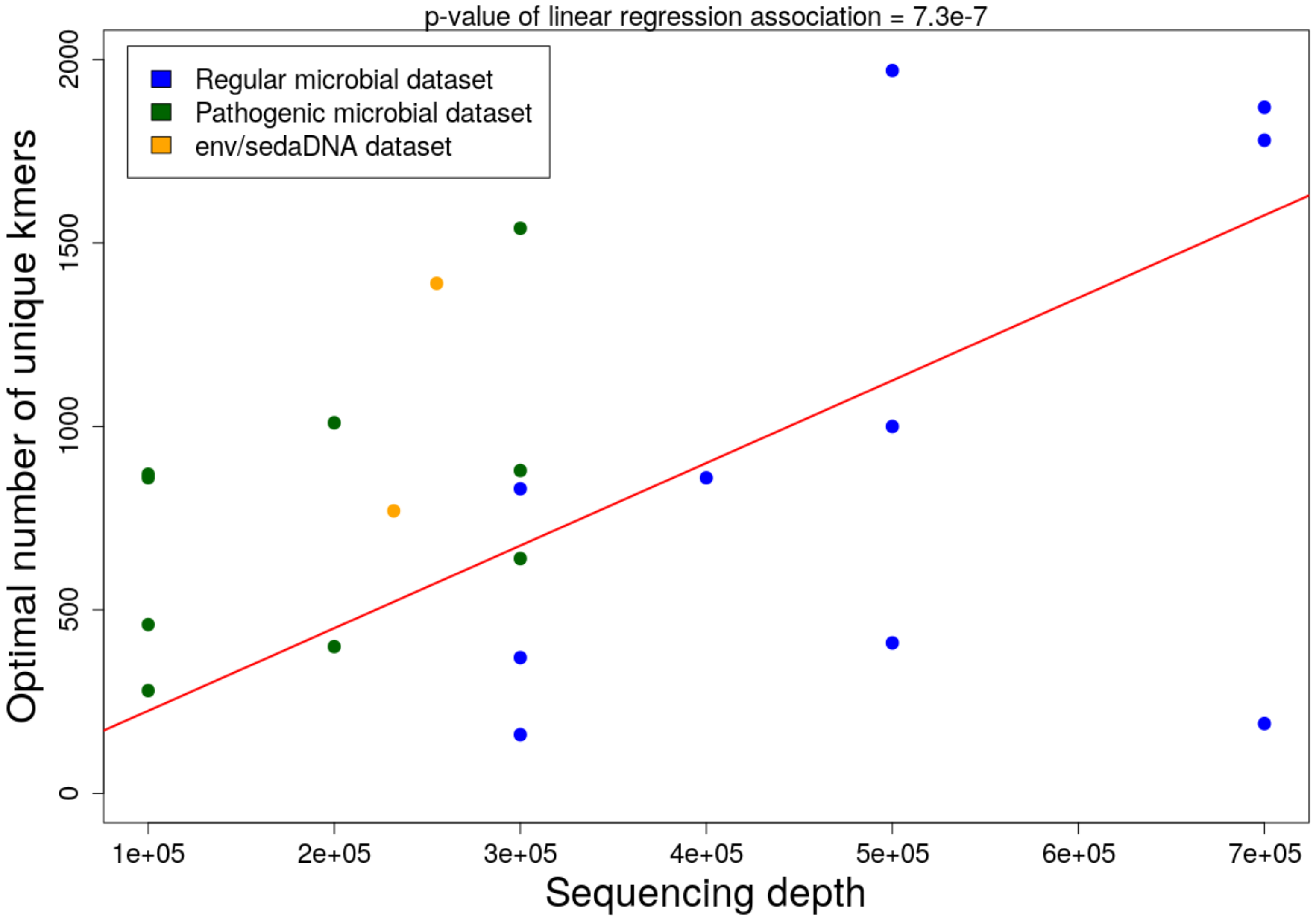

Supplementary Figure 8. Optimal number of unique k-mers grows approximately linearly with the sequencing depth following approximate law optimal_n_unique_kmers ~ 0.002 * seq_depth. Linear regression association $p=7*10^{-7}$, Pearson rho=0.4 and Spearman rho=0.3.

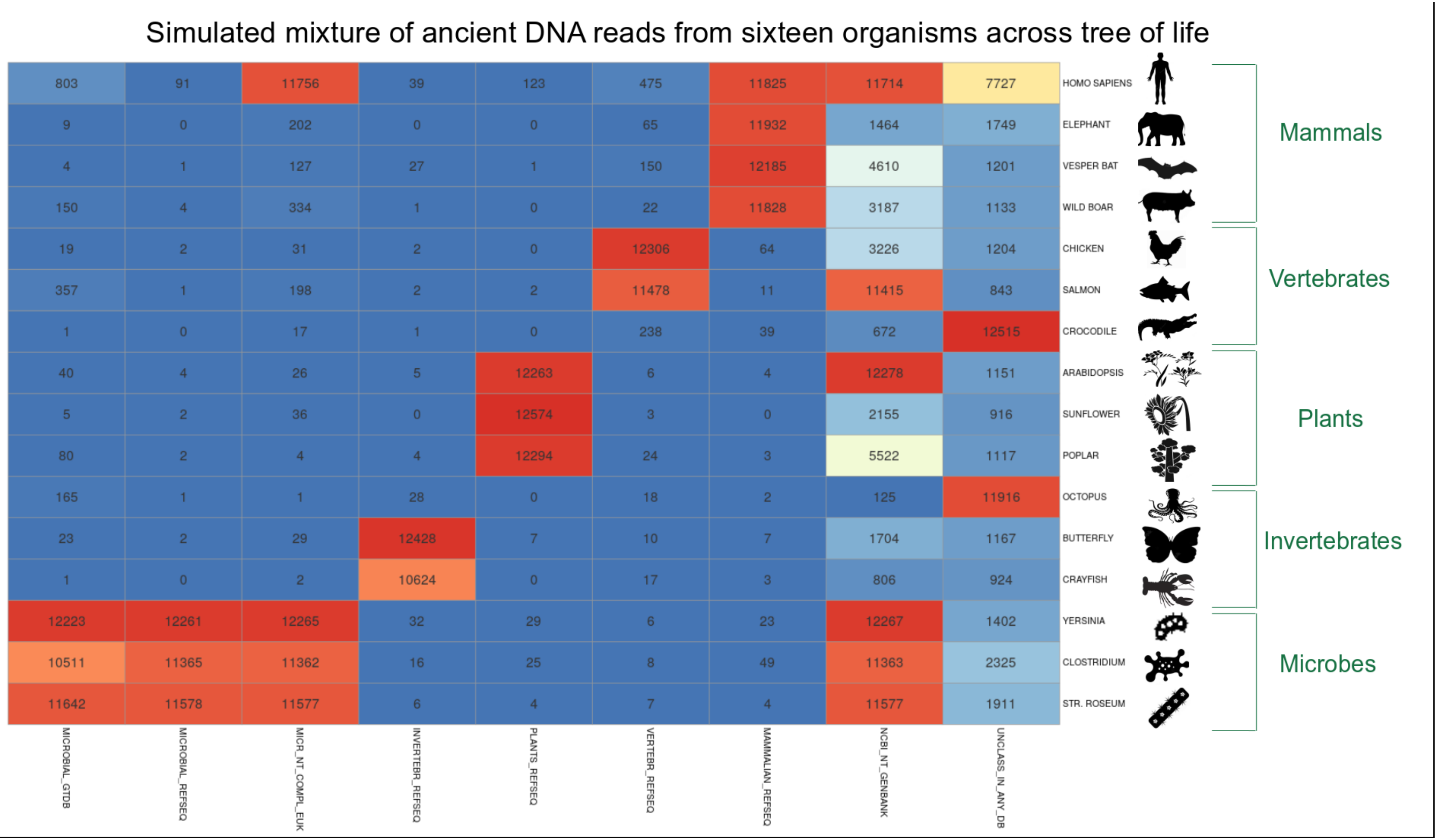

Supplementary Figure 9. Reconstruction of simulated ground truth for environmental / sedimentary metagenomic DNA samples with KrakenUniq using individual NCBI RefSeq databases (mammals, non-mammalian vertebrates, plants and microbes) and NCBI NT (GenBank) database. The counts represent the numbers of simulated reads assigned by KrakenUniq to each organism with different NCBI RefSeq and NT databases.

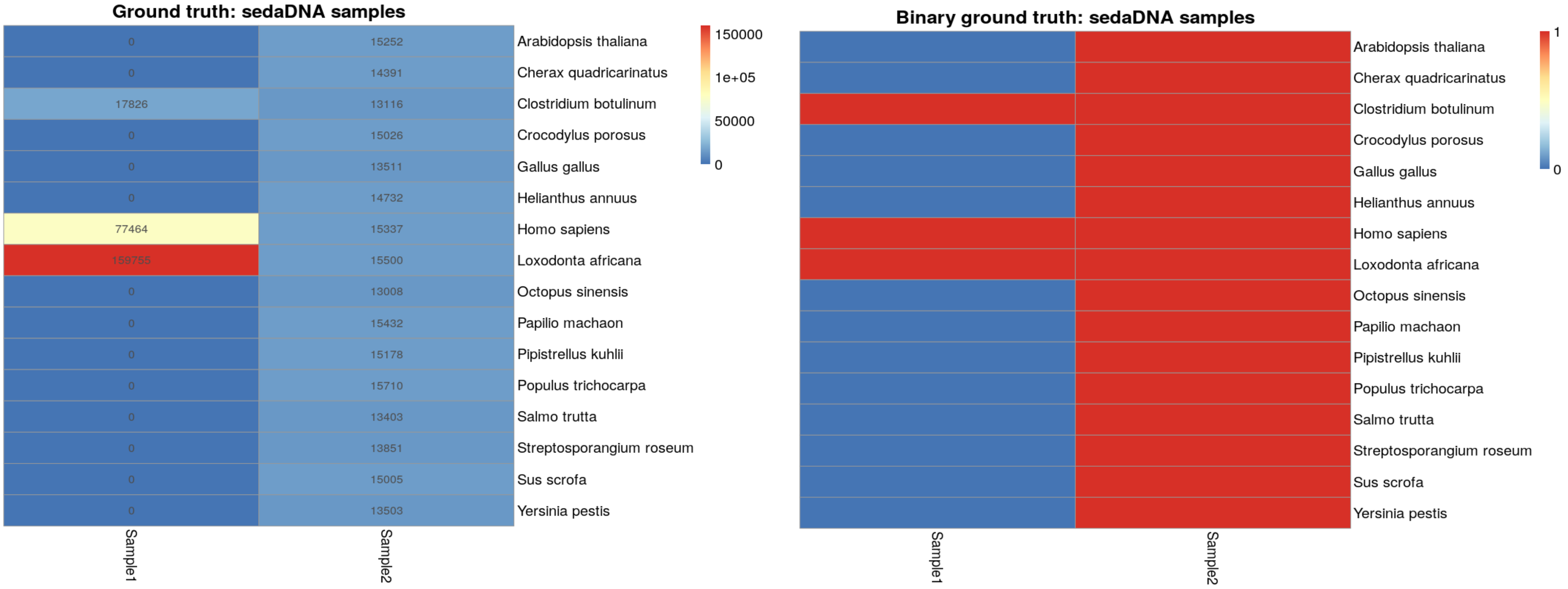

Supplementary Figure 10. Simulated with Gargammel ground truth of environmental / sedimentary ancient DNA samples: absolute simulated read counts (left), and corresponding binarized (presence vs. absence) representation (right).

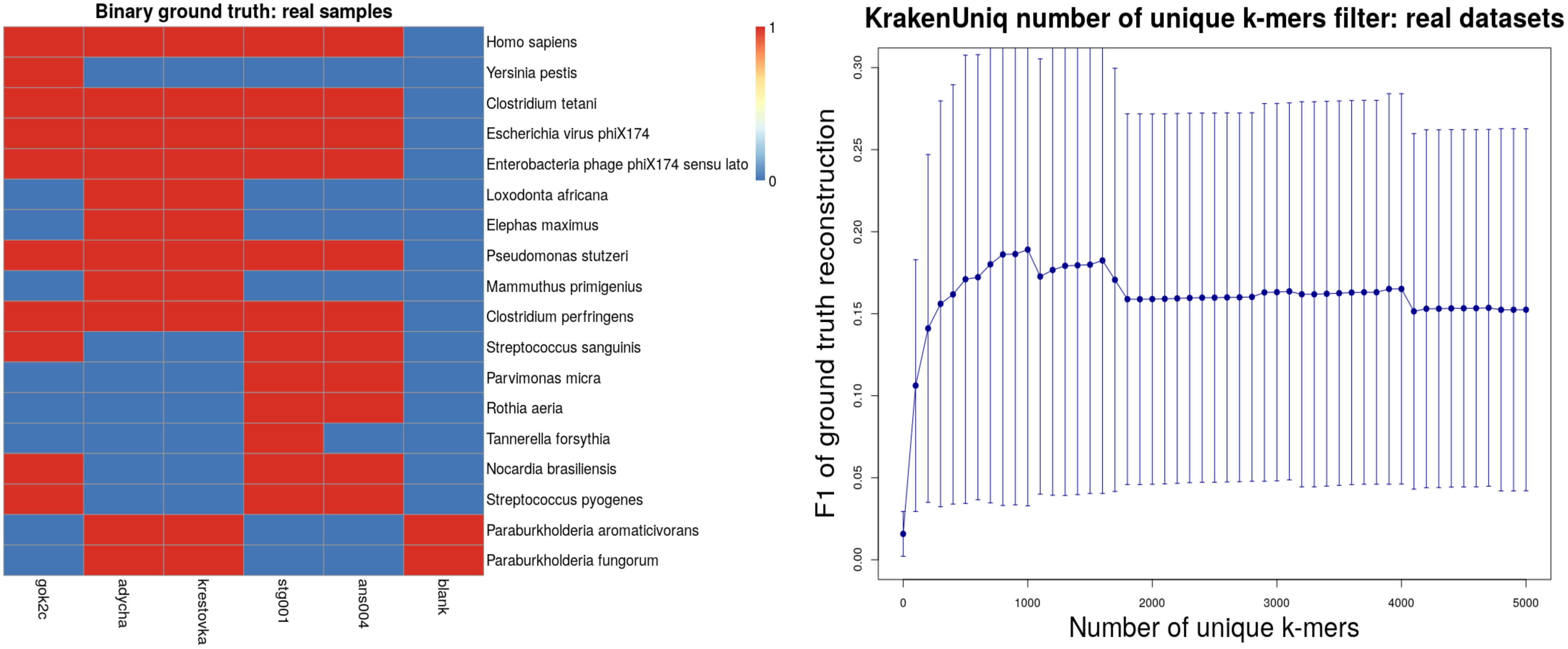

Supplementary Figure 11. Testing KrakenUniq number of unique *k*-mers filter on six real samples from three datasets [12, 18, 19]. The heatmap (left) presents organisms reported in ans004 and stg001 [12], gok2c [18], adycha, krestovka and blank [19] samples as ground truth. The F1-score, for different thresholds of the numbers of unique *k*-mers, was applied to quantify
